# Application of Soft Computing Techniques to Predict the Strength of Geopolymer Composites

**DOI:** 10.3390/polym14061074

**Published:** 2022-03-08

**Authors:** Qichen Wang, Waqas Ahmad, Ayaz Ahmad, Fahid Aslam, Abdullah Mohamed, Nikolai Ivanovich Vatin

**Affiliations:** 1 Department of Civil and Environmental Engineering, University of Iowa, Iowa City, IA 52242, USA; 2 Department of Civil Engineering, COMSATS University Islamabad, Abbottabad 22060, Pakistan; ayazahmad@cuiatd.edu.pk; 3 Faculty of Civil Engineering, Cracow University of Technology, 24 Warszawska Str., 31-155 Cracow, Poland; 4 Department of Civil Engineering, College of Engineering in Al-Kharj, Prince Sattam bin Abdulaziz University, Al-Kharj 11942, Saudi Arabia; f.aslam@psau.edu.sa; 5 Research Centre, Future University in Egypt, New Cairo 11745, Egypt; mohamed.a@fue.edu.eg; 6 Peter the Great St. Petersburg Polytechnic University, 195291 St. Petersburg, Russia; vatin@mail.ru

**Keywords:** geopolymer composites, sustainable materials, compressive strength, artificial intelligence, machine learning, prediction models

## Abstract

Geopolymers may be the best alternative to ordinary Portland cement because they are manufactured using waste materials enriched in aluminosilicate. Research on geopolymer composites is accelerating. However, considerable work, expense, and time are needed to cast, cure, and test specimens. The application of computational methods to the stated objective is critical for speedy and cost-effective research. In this study, supervised machine learning approaches were employed to predict the compressive strength of geopolymer composites. One individual machine learning approach, decision tree, and two ensembled machine learning approaches, AdaBoost and random forest, were used. The coefficient correlation (R^2^), statistical tests, and k-fold analysis were used to determine the validity and comparison of all models. It was discovered that ensembled machine learning techniques outperformed individual machine learning techniques in forecasting the compressive strength of geopolymer composites. However, the outcomes of the individual machine learning model were also within the acceptable limit. R^2^ values of 0.90, 0.90, and 0.83 were obtained for AdaBoost, random forest, and decision models, respectively. The models’ decreased error values, such as mean absolute error, mean absolute percentage error, and root-mean-square errors, further confirmed the ensembled machine learning techniques’ increased precision. Machine learning approaches will aid the building industry by providing quick and cost-effective methods for evaluating material properties.

## 1. Introduction

Cement-based conventional concrete (CBCC) is the most broadly utilized type of construction material on a global scale [1,2,3]. The primary constituents of CBCC are aggregates, water, and ordinary Portland cement (OPC) [4,5]. Following aluminum and steel, OPC is the third most energy-demanding substance on the earth, consuming 7% of the total energy of global industry [6,7]. Regrettably, the manufacture of OPC produces large quantities of greenhouse gases, i.e., CO_2_, which substantially add to climate change [8,9,10]. The production of OPC is anticipated to release 1.35 billion tons of greenhouse emissions annually [11,12,13]. Thus, scholars have focused their attempts on minimizing OPC usage through the use of alternate binder types. Alternatives to CBCC may include alkali-activated compounds such as geopolymers [14,15,16]. When precursors and activators react, alkali-activated compounds are formed. They have been categorized into two kinds based on the calcium proportion of the products formed during the reaction: those that are calcium-rich, with a Ca/(Si+Al) fraction above 1, and those that are calcium-deficient, i.e., geopolymers [17,18,19].

**Figure 1:**
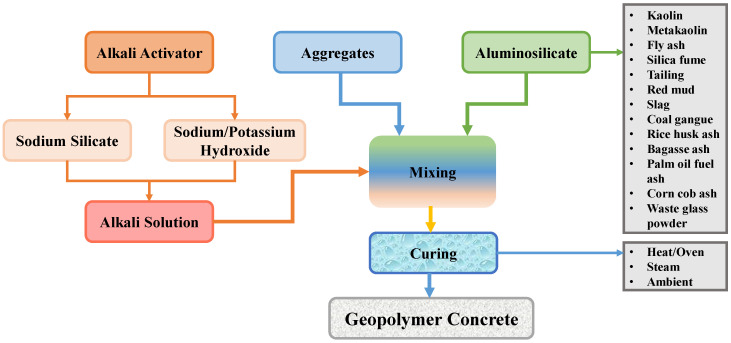
Production process of geopolymer concrete.

Geopolymer is a novel binder that was established to substitute OPC in concrete production [20,21,22]. The purpose is to acquire a building material that is free of OPC, environmentally caring, and sustainable. As industry and people grow, a significant amount of waste material (waste glass powder, fly ash, sugarcane bagasse ash, ground granulated blast furnace slag (GGBS), silica fume, and rice husk ash, for example) is generated and disposed of in landfills. Due to the fact that these waste products contribute to pollution, their disposal in landfills is dangerous to the ecosystem [23,24,25,26]. Since geopolymer composites (GPCs) require raw ingredients with a high aluminosilicate content, which are found in these waste materials, recycling these waste materials can help to reduce environmental pollution [27,28,29,30]. The method of producing GPC is represented in Figure 1, along with the various components and curing regimes employed. Consumption of these waste materials benefits both the environment and the economy, as the demand for inexpensive housing will increase as the population expands [31,32,33]. GPC has been the topic of broad research and development on a global scale, and it may one day become the best green construction material [34,35,36,37]. GPC, on the other hand, has the potential to make a significant contribution to the prolonged sustainability of both CBCC technology and the building sector.

Artificial intelligence (AI) advancements have resulted in the widespread usage of machine learning (ML) techniques for anticipating the properties of a variety of materials [38,39,40,41]. Ahmad et al. [14] conducted a comparative investigation of three ML approaches for assessing the compressive strength (CS) of fly ash-based GPC, including decision tree (DT), AdaBoost, and bagging regressor (BR). It was noted that the BR model was the most precise of the models examined. Ahmad et al. [42] forecasted the CS of concrete, including recycled coarse aggregate using an artificial neural network (ANN) and gene expression programming (GEP). The GEP technique was found to be more predictively exact than the ANN technique. Song et al. [43] used an ANN method to estimate the CS of concrete, and they observed a satisfactory forecasting ability. Nguyen et al. [44] predicted the CS and tensile strength of high-performance concrete using a range of ML approaches. They concluded that ensembled ML techniques outperformed individual ML techniques in terms of precision. Thus, numerous scientists have reported on diverse ML strategies that improve the accuracy of material property estimation. As a result, it is critical to conduct additional in-depth investigations to elucidate this topic.

This research concentrates on the use of ML approaches to estimate the CS of GPCs. Three different types of ML approaches were used, including decision tree (DT), AdaBoost, and random forest (RF), and their performance was assessed using statistical tests and correlation coefficient (R^2^). Additionally, the validity of each strategy was determined using k-fold analysis and error distributions. DT is an individual ML technique, whereas AdaBoost and RF are ensemble ML algorithms. This research is novel in that it estimates the CS of GPC using both individual and ensembled ML techniques, whereas experimental investigations need significant human work, experimental costs, and time for material acquisition, casting, curing, and testing. The use of modern techniques, like ML, in the field of civil engineering to predict material properties will reduce human effort and save time since experimental work for said purpose can be eliminated. ML approaches require a data set that might be retrieved from the literature as considerable research has been conducted to experiment with the material properties, and the data set can be used to train the ML models and estimate the various characteristics of a material. This study aims to identify the most suitable ML technique for the CS of GPCs in terms of results prediction and the influence of input parameters on the model’s performance.

## 2. Methods

### 2.1. Description of Data

**Figure 2:**
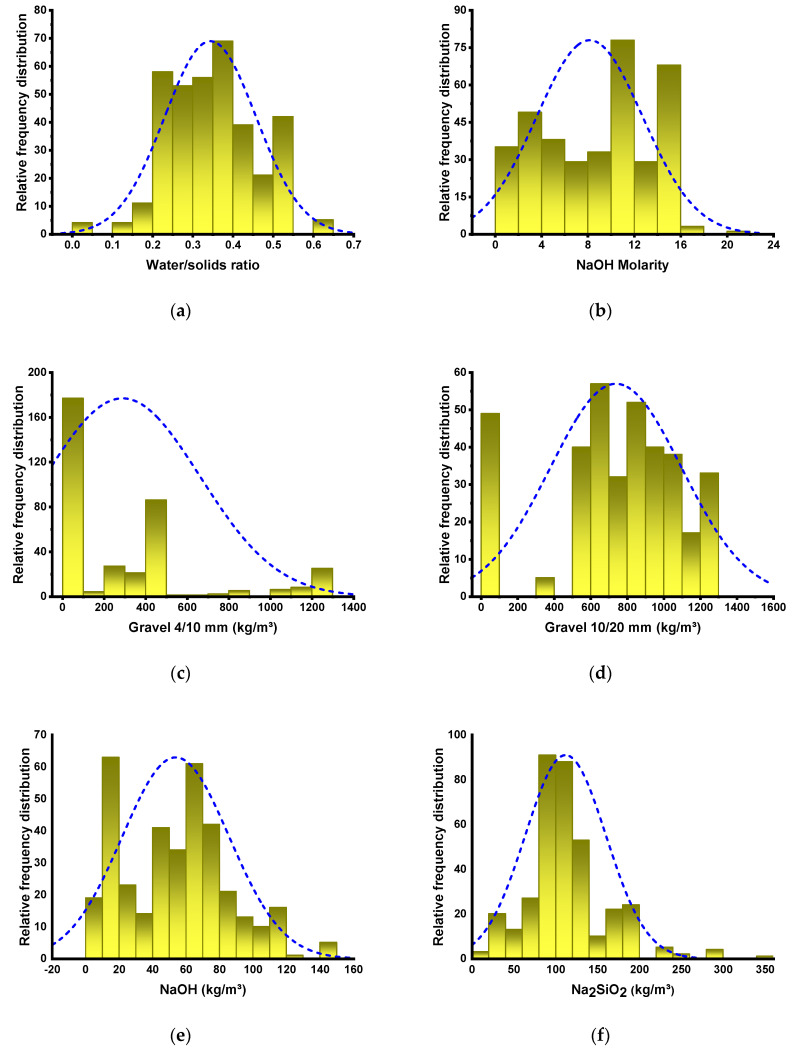

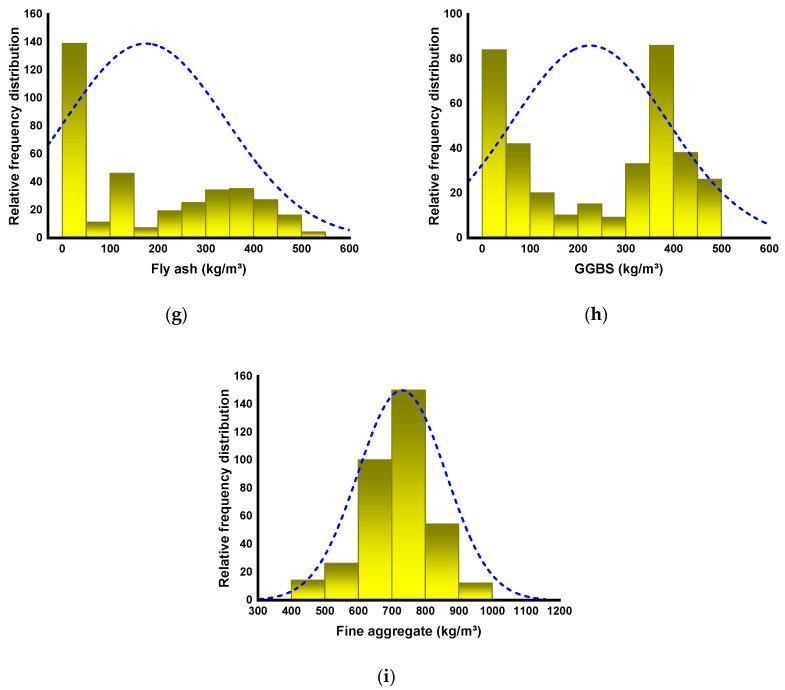
Relative frequency dispersal of inputs: (**a**) Water/solids ratio; (**b**) NaOH Molarity; (**c**) Gravel 4/10 mm; (**d**) Gravel 10/20 mm; (**e**) NaOH; (**f**) Na_2_SiO_3_; (**g**) Fly ash; (**h**) GGBS; (**i**) Fine aggregate.

To obtain an appropriate result, SML algorithms require a varied range of input variables [45,46,47]. The CS of GPC was estimated using data retrieved from the literature (attached as a Supplementary File). To prevent bias representation, experimental data were randomly chosen from the literature. The literature published on the use of comparable materials for the CS of GPC was assessed. While most papers examined additional features of GPC, this study acquired CS-based data points to run the algorithms. Nine variables were included as inputs in the algorithms, containing water/solids ratio, NaOH molarity, gravel 4/10 mm, gravel 10/20 mm, NaOH, Na_2_SiO_3_, fly ash, GGBS, and fine aggregate, with CS as the output variable. The number of inputs and datasets have a considerable effect on the model’s outcome [48,49,50]. In the current investigation, 363 data points were used to run ML algorithms. Table 1 summarizes the descriptive statistic evaluation of each input variable. The term “descriptive statistics” refers to a group of concise, factual measurements that generate an outcome, which may be the whole population or a subset of the population. The mean, median, and mode variables represent fundamental tendency, whereas the maximum, minimum, and standard deviation represent variability. The table provides all the mathematical terms for the model’s input variables. The relative frequency distribution of all variables used in the analysis is depicted in Figure 2. It depicts the total number of interpretations related to each value or combination of values. It is intrinsically related to probability dispersal, a widely used statistical term.

### 2.2. Machine Learning Algorithms Employed

**Figure 3:**
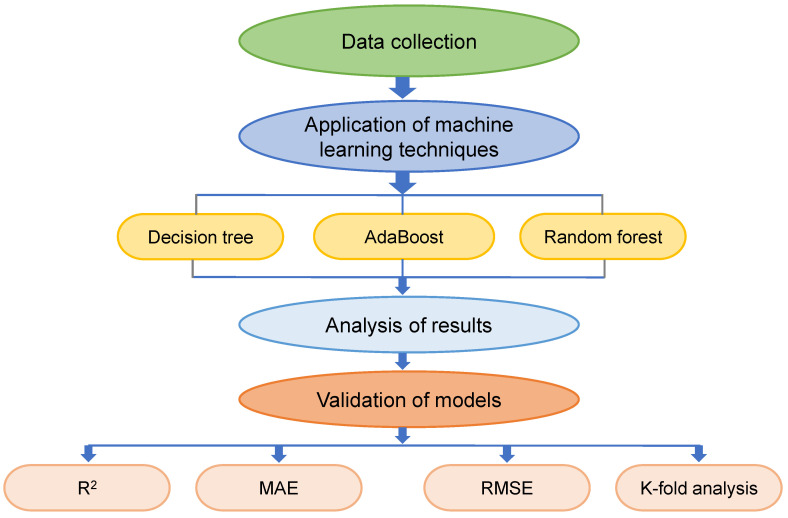
Flowchart of research methodology.

Individual ML approaches (DT) and ensemble ML techniques (AdaBoost and RF) were used to accomplish the study’s objectives, with Python scripting via the Anaconda Navigator package. To run DT, AdaBoost, and RF models, the tool Spyder (version 4.3.5) was chosen. These algorithms are often used to anticipate desired outcomes based on input variables. These algorithms, among other things, are capable of forecasting the temperature effect, strength properties, and durability of materials [51,52]. Throughout the modeling phase, nine input variables and one output variable (i.e., CS) were employed. The R^2^ value for the projected result reflected the validity/precision of all models. The R^2^ represents the extent of divergence; a value close to zero suggests higher divergence, but a value close to one shows that the model and data are almost completely suited [14]. The sub-sections below detail the ML techniques applied in this research. Additionally, to validate models, statistical and k-fold analysis and error assessments are carried out on all techniques, involving root-mean-square error (RMSE), mean absolute percentage error (MAPE) and mean absolute error (MAE). Moreover, sensitivity analysis is employed to discover the effect of each input parameter on the outcome estimation. The flowchart in Figure 3 depicts the research strategy.

#### 2.2.1. Decision Tree

**Figure 4:**
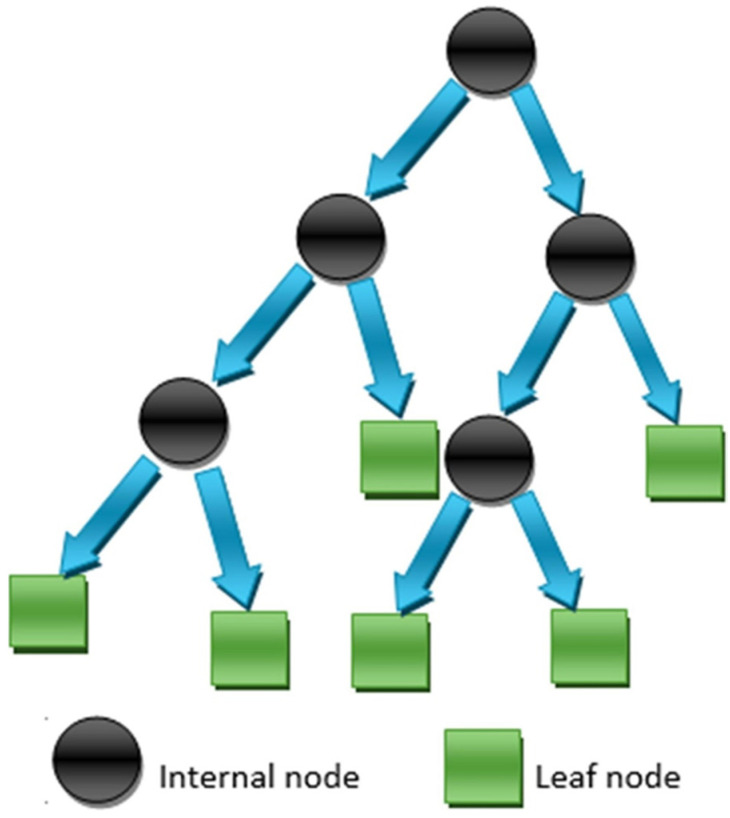
Decision tree schematic representation [14]. Reprinted with permission from ref. [14]. Copyright 2022 Elsevier B.V.

DTs are created by the development of algorithms that divide a dataset into branch-like portions. These portions combine to create an upturned tree, which begins with a root node at the top [53]. Figure 4 demonstrates an illustration of such a tree with five nodes and six leaves. As seen in the figure, a DT tree can contain both uninterrupted and isolated features. Correlations between the object of analysis and the input fields are used to produce the decision rule for branching or segmenting underneath the root node. After establishing the link, one or more decision rules specifying the relationships between the inputs and targets can be produced. Decision rules reliably estimate the values of new or unknown observations that include input values but not targets. The errors are computed at each division point, and the variable with the lowest fitness function value is chosen as a split point, followed by the procedure for the other variables.

#### 2.2.2. AdaBoost

**Figure 5:**
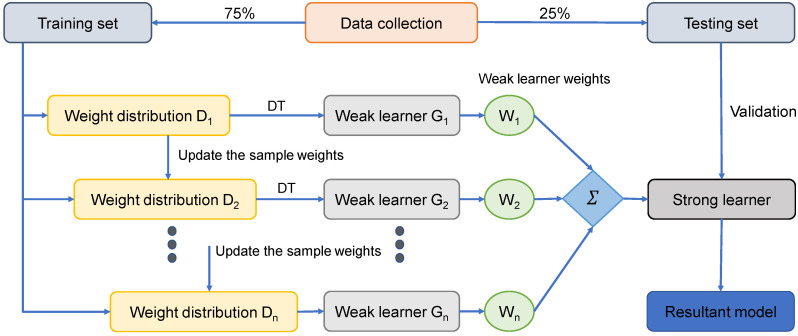
Schematic representation of AdaBoost technique.

The AdaBoost approach is the most often used ensembled ML algorithm from the boosting group of ensembled ML techniques. AdaBoost’s distinguishing characteristic is that it utilizes the initial training data to construct a weak learner, after which it modifies its distribution of training data depending on its projection performance in the following turn of weak learner training. Remember that the training samples with less forecast precision from the previous stage will be given more attention in the following phase. After that, the weak learners are then coupled with a strong learner by applying a variety of weights to form a final combination [39]. AdaBoost is simple to implement. In general, it consists of four stages: (i) data collection; (ii) development of a strong learner; (iii) testing or confirmation of the learner; and (iv) use of the learner for engineering challenges. Clearly, the second step is important to the AdaBoost algorithm. As mentioned previously, it consists of two components: a framework for integrating weak learners into a strong one and a regression learning algorithm for producing the weak learner from the training data. The weak learner is generated using the decision tree (DT) algorithm [39], and the weak learners are combined using the median of the weighted weak learners. Figure 5 illustrates the flow diagram for this technique.

#### 2.2.3. Random Forest

**Figure 6:**
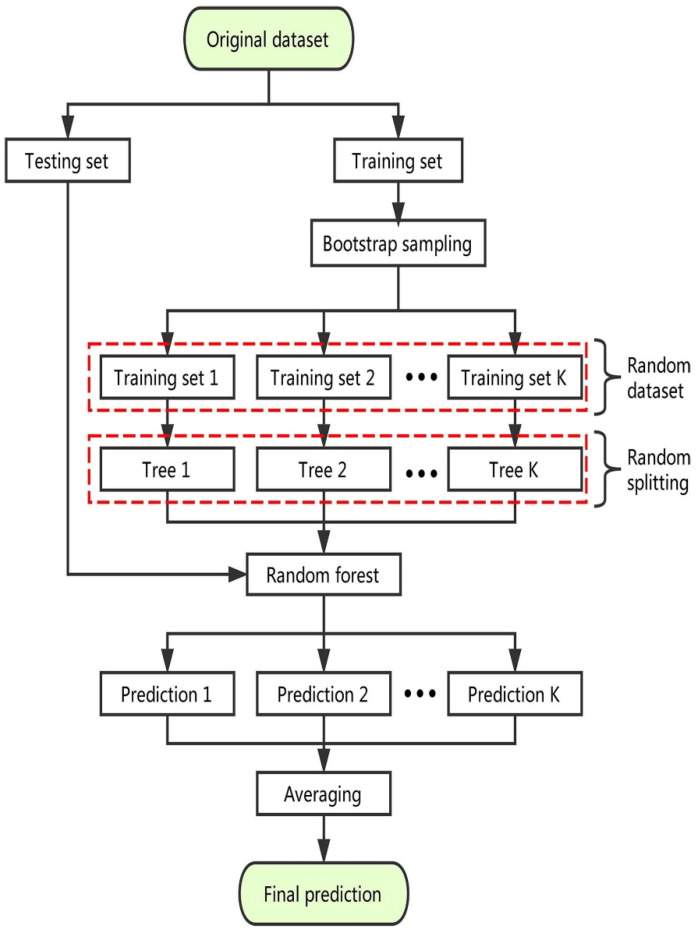
Schematic representation of random forest algorithm [54]. Reprinted with permission from ref. [54]. Copyright 2019 Elsevier Ltd.

RF is implemented via the random split selection on bagging DTs [54]. Figure 6 illustrates the production and process of the RF model schematically. Every tree in the forest is constructed using a randomly chosen training set, and each split within each tree is built using a randomly selected subset of input variables, resulting in a forest of trees [55]. The addition of this unpredictability boosts the tree’s diversity. The forest is entirely composed of fully-grown binary trees. The RF method has been exceedingly effective as a common-purpose classification and regression tool. The technique, which combines the predictions of several randomized DTs, has shown higher precision in circumstances where the quantity of variables exceeds the quantity of observations. Moreover, it is adaptive to both large-scale and ad hoc learning tasks, returning metrics of different importance [54].

## 3. Models Results

### 3.1. Decision Tree Model

**Figure 7:**
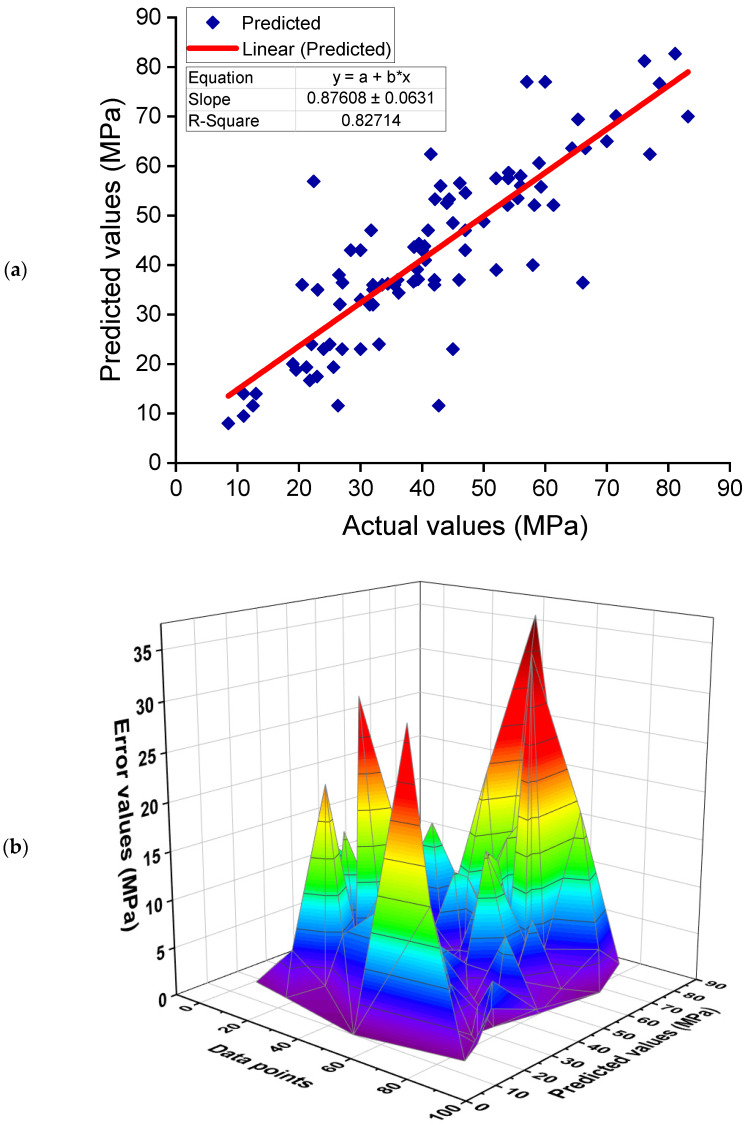
Decision tree model: (**a**) correlation between experimental and estimated results; (**b**) dispersal of predicted and error values.

Figure 7 illustrates the results of the DT model for the CS of GPC. Figure 7a shows the link between experimental and projected outcomes. The DT technique generated results with a satisfactory level of accuracy and a small disparity between experimental and predicted outcomes. The R^2^ of 0.83 confirms the satisfactory performance of the DT model in forecasting the CS of GPC. The dispersion of predicted and error values for the DT model is represented in Figure 7b. The error values were analyzed, and it was determined that the minimum, average, and highest values were 0.00, 7.02, and 36.59 MPa, respectively. Additionally, the percentage distribution of error values was determined, and it was discovered that 37.4% of values were less than 3 MPa, 38.5% were between 3 and 10 MPa, and only 24.2% were above 10 MPa. Additionally, the dispersion of error values implies that the DT model performs satisfactorily.

### 3.2. AdaBoost Model

**Figure 8:**
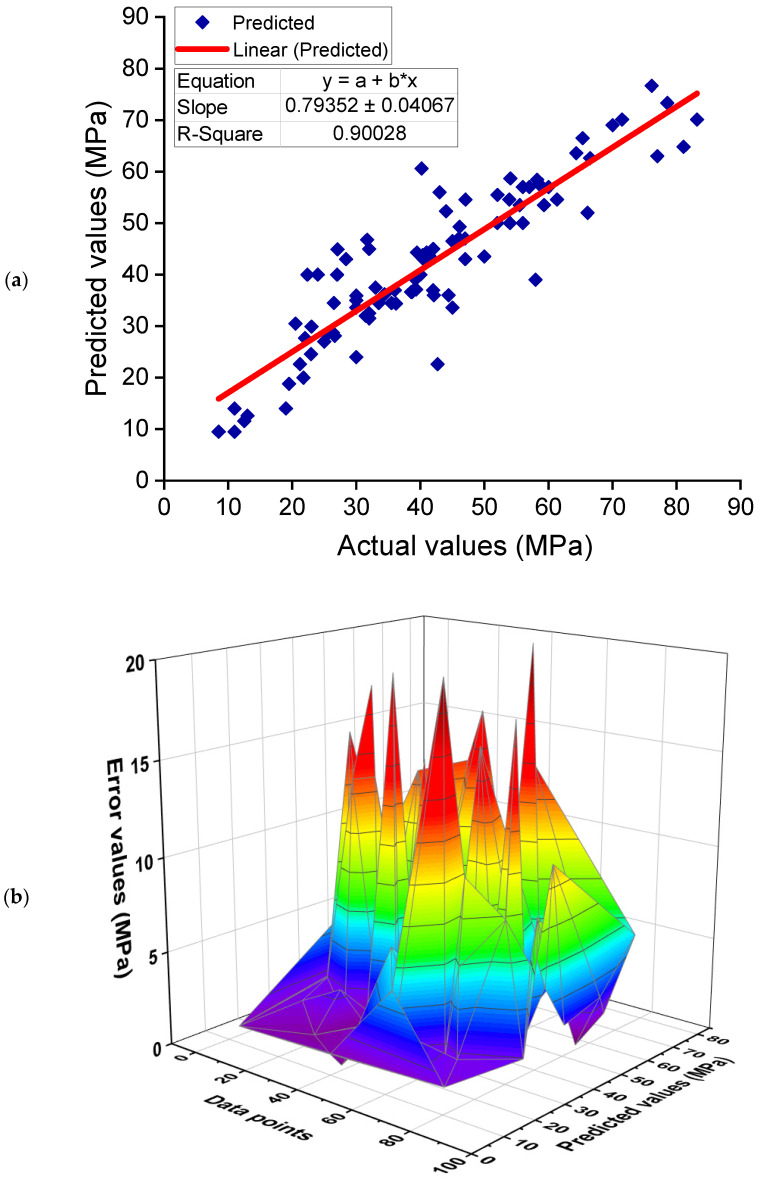
AdaBoost model: (**a**) correlation between experimental and estimated results; (**b**) dispersal of predicted and error values.

Figure 8 depicts the AdaBoost model’s findings for estimating the CS of GPC. The relationship between experimental and anticipated outcomes is depicted in Figure 8a. The AdaBoost approach produced output with higher precision and the least amount of divergence between actual and anticipated outcomes. With an R^2^ of 0.90, the AdaBoost model is quite accurate at forecasting the CS of GPC. Figure 8b illustrates the dispersion of predicted and error values for the AdaBoost model. The training set’s lowest, average, and maximum error values were determined to be 0.00, 5.20, and 20.40 MPa, respectively. The error distribution was 46.2%less than 3 MPa, 35.2% between 3 and 10 MPa, and only 18.7% greater than 10 MPa. The distribution of error values indicates the AdaBoost model’s higher precision in forecasting outcomes.

### 3.3. Random Forest Model

**Figure 9:**
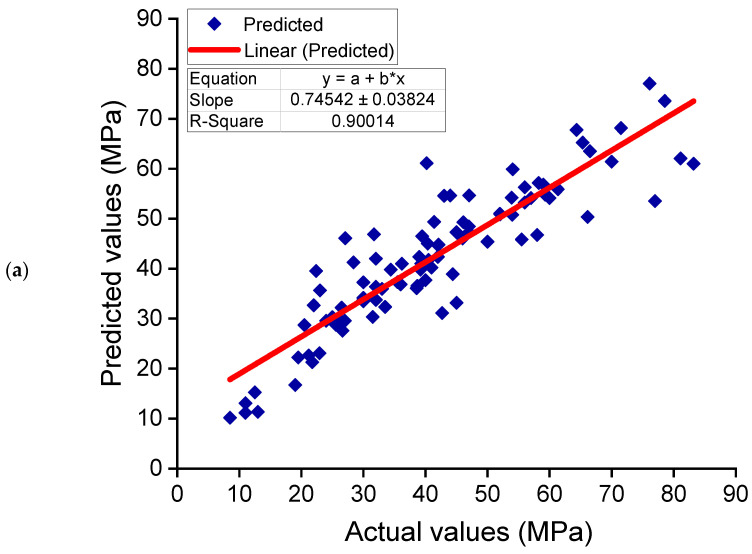

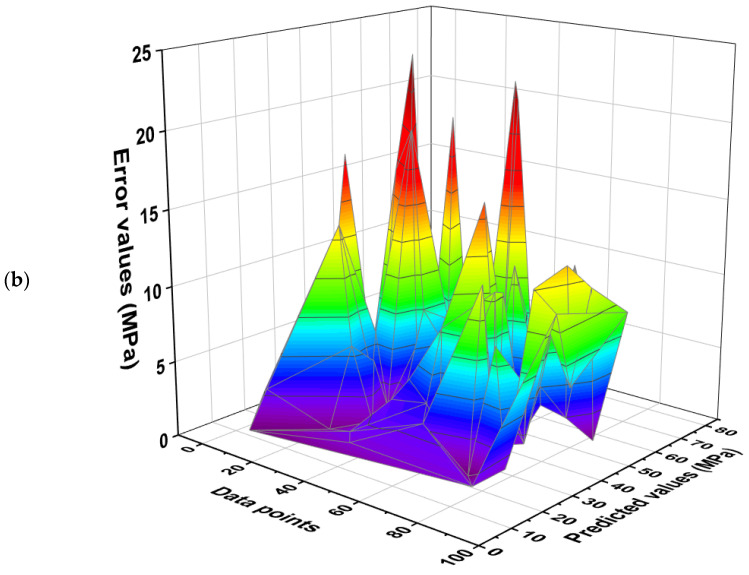
Random forest model: (**a**) correlation between experimental and estimated results; (**b**) dispersal of predicted and error values.

Figure 9a,b demonstrate an assessment of the RF model’s experimental and estimated results. Figure 9a depicts the link between experimental and estimated findings, with an R^2^ of 0.90 signifying that the RF model has a comparable precision to the AdaBoost model in estimating the GPCs CS. Figure 9b shows the dispersal of experimental, expected, and error values for the RF model. The lowest, average, and highest error values were determined to be 0.06, 5.33, and 23.45 MPa, respectively. The error distribution was 47.3% less than 3 MPa, 34.1% between 3 and 10 MPa, and only 18.7% larger than 10 MPa. These reduced error values demonstrate the RF model’s higher exactness than the DT model and similar accuracy to the AdaBoost model.

## 4. Validation of Models

**Figure 10:**
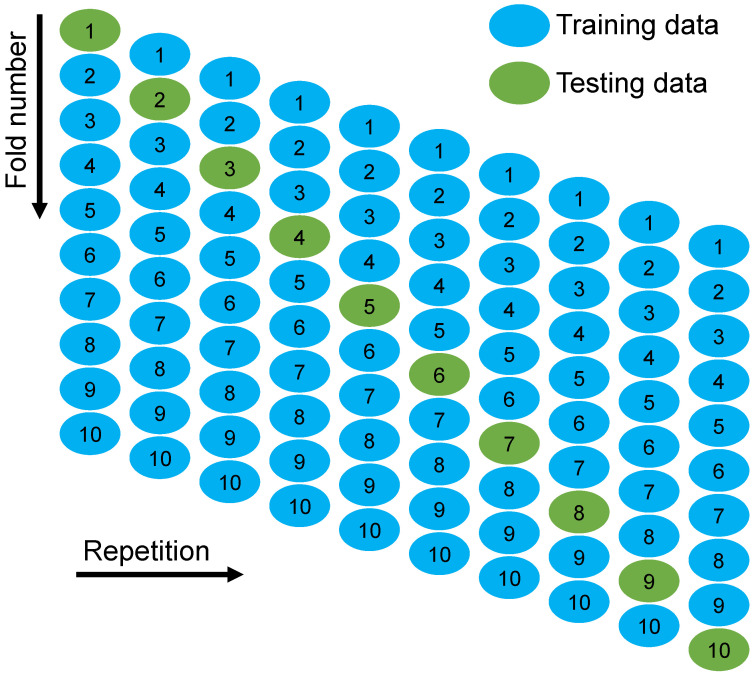
K-fold cross-validation procedure.

Statistical and k-fold analysis approaches were used to validate the models. The k-fold technique is frequently used to ascertain a technique’s validity [42], during which relevant data are arbitrarily scattered and divided into 10 classes. As seen in Figure 10, nine groups will be used to train the model, while one group will be utilized to validate it. Approximately 75% of the data was utilized for training the models, whereas 25% was utilized to assess the models that were employed. When the errors (MAE and RMSE) are low and the R^2^ value is high, the model is more accurate. In addition, the operation ought to be reiterated ten times to achieve a reasonable conclusion. This extensive effort contributes significantly to the model’s remarkable accuracy. Furthermore, as shown in Table 2, all models were statistically evaluated in terms of errors (MAE, MAPE, and RMSE). These assessments also confirmed the AdaBoost and RF model’s higher accuracy as a result of their lower error readings when compared to the DT model. The predictive performance of the techniques was determined statistically using Equations (1)–(3), which were acquired from previous studies [38,56,57].

(1) $$\mathrm{MAE}=\frac{1}{n}\overset{n}{\underset{i=1}{\sum }}\left|P_{i}-T_{i}\right|$$

(2) $$\mathrm{RMSE}=\sqrt{\sum \frac{{\left(P_{i}-T_{i}\right)}^{2}}{n}}$$

(3) $$\mathrm{MAPE}=\frac{100\%}{n}\overset{n}{\underset{i=1}{\sum }}\frac{\left|P_{i}-T_{i}\right|}{T_{i}}$$

where n = sum of data samples, $P_{i}\;$= predicted values, and $T_{i}$ = experimental values from the data set.

In order to figure out how well the k-fold cross-validation worked, MAE, MAPE, RMSE, and R^2^ were calculated, and their values are provided in Table 3. The DT model’s MAE values ranged from 7.02 to 19.78 MPa, with an average of 11.08 MPa. When comparing, the MAE values for the AdaBoost model ranged between 5.20 and 14.68 MPa, with an average of 8.68 MPa. As for the AdaBoost model, the MAE values were between 5.33 and 18.47 MPa, with an average of 8.97 MPa. Similarly, the average MAPE for DT, AdaBoost, and RF models was noted to be 17.04%, 13.16%, and 13.47%, respectively. The average RMSE values for the DT, AdaBoost, and RF models were 15.89, 11.18, and 11.94 MPa. On the other hand, the average R^2^ values for DT, AdaBoost, and RF models were 0.59, 0.67, and 0.65, respectively. The AdaBoost and RF models with the lower error values and the higher R^2^ values are more accurate in forecasting the CS of GPC when compared to the DT model.

## 5. Sensitivity Analysis

**Figure 11:**
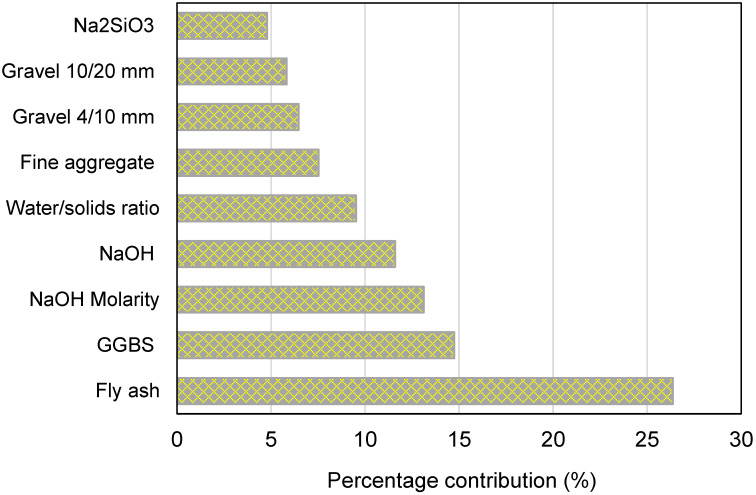
Input variables’ contributions to predicting outcomes.

The intention of this assessment is to ascertain the effect of input parameters on GPC’s CS predicting. The expected outcome is greatly affected by the input variables [14]. Figure 11 demonstrates the impact of the inputs on the CS estimate of GPC. The investigation determined that fly ash was the most important constituent, accounting for 26.37% of the total, followed by GGBS at 14.74% and NaOH molarity at 13.12%. The remaining input variables, on the other hand, contributed less to the forecast of GPC’s CS, with NaOH accounting for 11.60%, the water/solids ratio accounting for 9.52%, fine aggregate accounting for 7.53%, gravel 4/10 mm accounting for 6.48%, gravel 10/20 mm accounting for 5.84%, and Na_2_SiO_3_ accounting for 4.80%. Sensitivity analysis generated outcomes related to the number of input parameters and data points employed to construct the models. The influence of an input parameter on the technique’s output was determined using Equations (4) and (5).

(4) $$N_{i}=f_{\max}\left(x_{i}\right)-f_{\min}\left(x_{i}\right)$$

(5) $$S_{i}=\frac{N_{i}}{\sum_{j-i}^{n}N_{j}}$$

where $f_{\max}\left(x_{i}\right)$ and $f_{\min}\left(x_{i}\right)$ are the peak and bottom of the expected result on the ith output, respectively, whereas other input variables are maintained constant at their mean values. $S_{i}$ is the achieved contribution proportion for a particular variable.

## 6. Discussions

### 6.1. Comparison of Machine Learning Models

**Figure 12:**
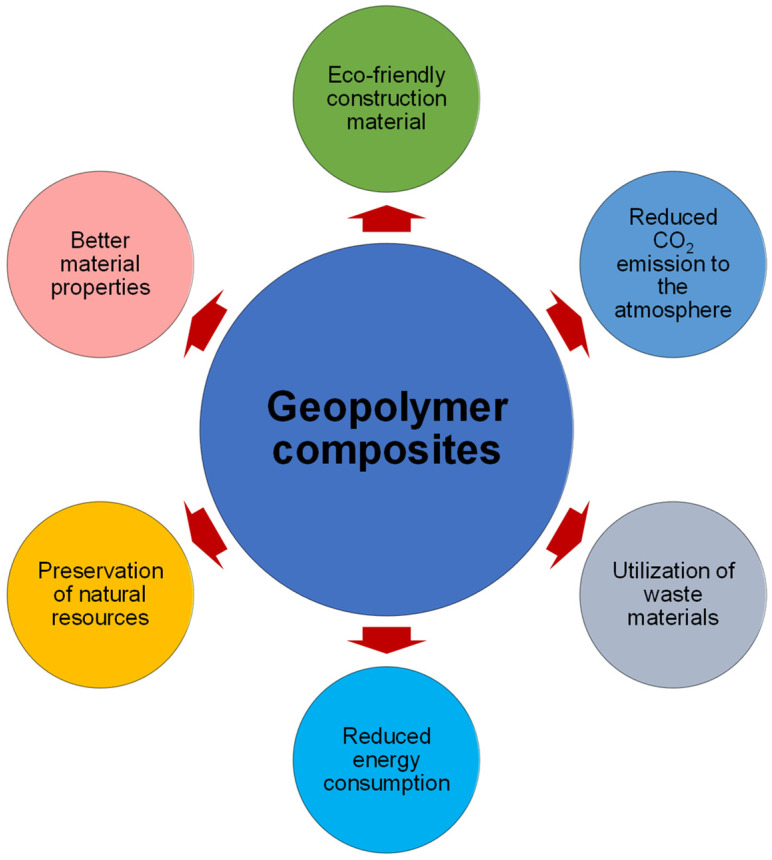
Advantages of geopolymer composites produced with waste materials.

The objective of this study was to contribute to the existing study area regarding the implementation of contemporary approaches for estimating the CS of GPC. This type of research will aid the building industry by developing rapid and cost-effective solutions for material property prediction. Additionally, by utilizing these strategies to promote eco-friendly construction, the adoption and use of GPC in construction will be accelerated. Since GPC may be made from waste materials containing aluminosilicates, its use in construction will have a number of advantages, as seen in Figure 12. This study demonstrates how ML methods can be employed to anticipate the CS of GPC. Three ML techniques were used in the study: one individual (DT) and two ensembled (AdaBoost and RF). Each technique was examined for accuracy in order to discover which is the most efficient predictor. The AdaBoost and RF models produced more exact results with an R^2^ of 0.90, compared to the DT model, which yielded an R^2^ of 0.83.

Furthermore, all models’ accuracy was validated using the statistical k-fold analysis approach. The fewer error values in the model, the more precise it is. The higher accuracy of AdaBoost and RF models towards the prediction of outcomes is also reported by other researchers [39,58,59]. Feng et al. [39] noticed the superior performance of the AdaBoost model compared to individual models, including ANN and support vector machine (SVM), based on higher R^2^ and lower error values. Similarly, Farooq et al. [59] compared the performance of RF with ANN, GEP, and DT techniques and reported the higher precision of the RF model than the others with an R^2^ of 0.96. However, determining and recommending the optimal ML model for forecasting outcomes through a variety of areas is complicated, as the performance of a model is greatly reliant on the input parameters and quantity of data points utilized to execute the algorithm. The previous studies concluded that up to 300 data points and a minimum of 8 input variables could result in the higher precision of the ML models [56,60]. Hence, the data set retrieved for the current investigation is suitable for the ML model’s best performance.

**Figure 13:**
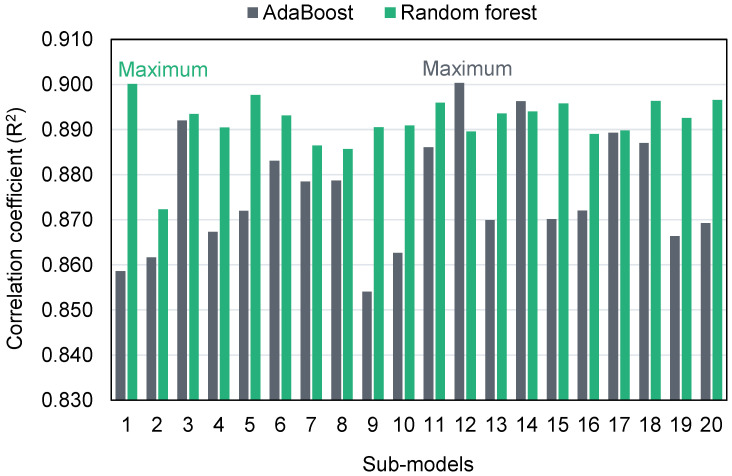
Coefficient of determination of sub-models.

The ensembled ML algorithms commonly exploit the weak learner by generating sub-models that may be trained on data and adjusted to optimize the R^2^ value. The dispersion of R^2^ values for the AdaBoost and RF sub-models is shown in Figure 13. The minimum, average, and highest R^2^ values for AdaBoost sub-models were 0.854, 0.876, and 0.900, respectively. Similarly, the minimum, average, and highest R^2^ values for RF sub-models were 0.872, 0.892, and 0.900, respectively. These results demonstrate that both the AdaBoost and RF sub-models have comparable values and a high degree of precision in forecasting GPC’s CS. Additionally, a sensitivity analysis was done to ascertain the effect of all inputs on the expected CS of GPC. The model’s performance might be affected by the input parameters and the dataset’s size. The sensitivity analysis determined how each of the nine input characteristics contributed to the projected output. Fly ash, GGBS, and NaOH molarity were determined to be the three most significant input variables.

### 6.2. Comparison of Experimental and Predicted Results

**Figure 14:**
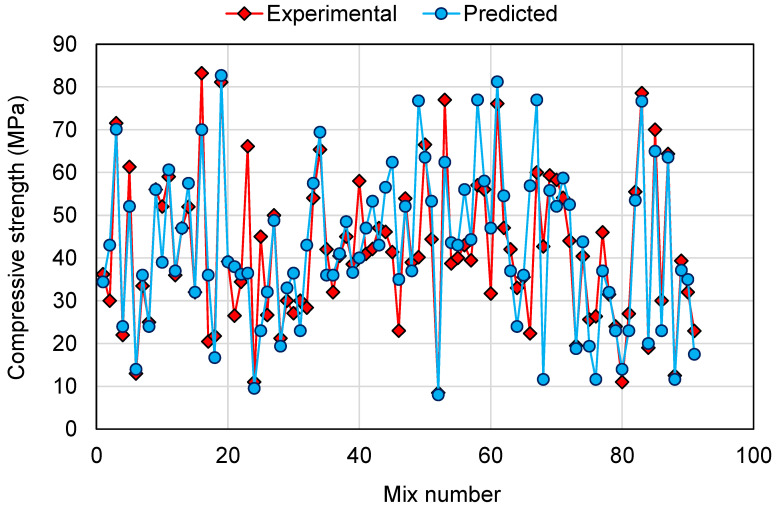
Comparison of experimental and predicted results for the decision tree model.

**Figure 15:**
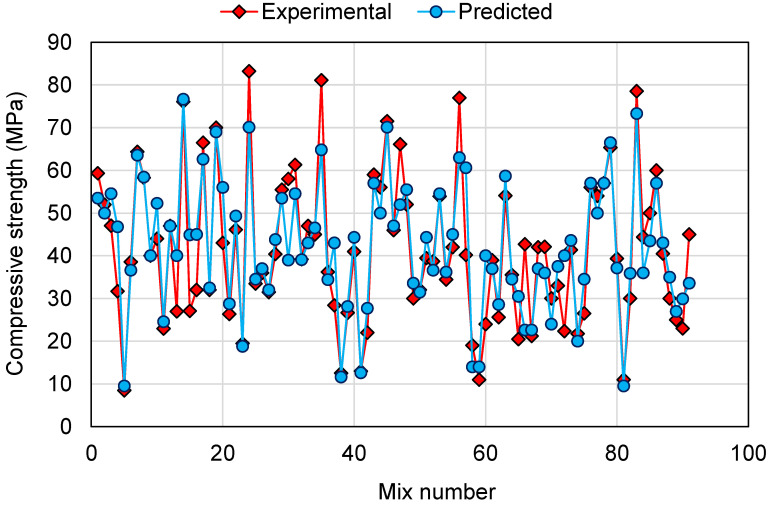
Comparison of experimental and predicted results for the AdaBoost model.

**Figure 16:**
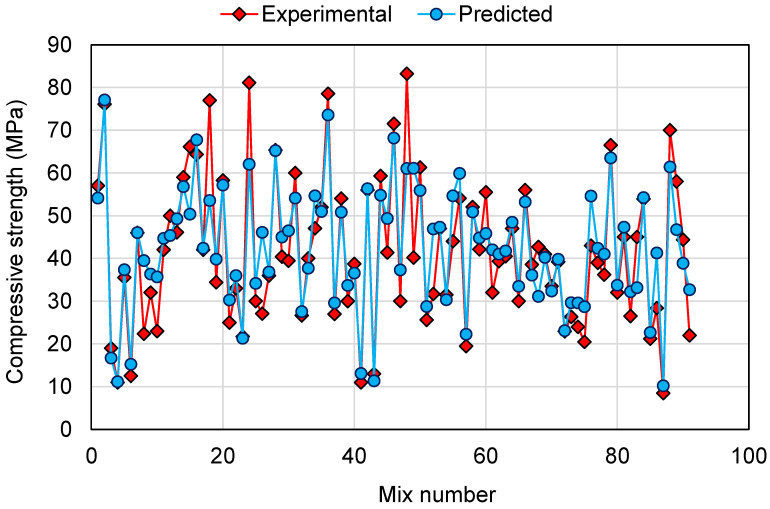
Comparison of experimental and predicted results for the random forest model.

To compare the experimental and predicted results for all the models employed in this study, Figure 14, Figure 15 and Figure 16 are generated for 91 mixes. The intention of this comparison was to determine the deviation of the predicted results from the experimental results for the validation of the employed models in estimating the CS of GPCs. This analysis revealed that for the DT model, the deviation from the experimental results was between 0.00 and 36.59 MPa, with an average of 7.02 MPa. Furthermore, for 34 mixes, the deviation from the experimental results was less than 3 MPa, from 3 to 10 MPa deviation was noted in 35 mixes, and above 10 MPa deviation was noted in 22 mixes (Figure 14). This showed a moderate deviation of the predicted results compared to the experimental results for the DT model. A similar comparison for the AdaBoost model revealed that the deviation of the results was in the range of 0.00 to 20.40 MPa with an average of 5.20 MPa. Additionally, for 42 mixes, the deviation was less than 3 MPa. Deviation from 3 to 10 MPa was observed in 32 mixes, and deviation greater than 10 MPa was observed in only 17 mixes (Figure 15). This showed the higher precision of the AdaBoost model compared to the DT. Similarly, the RF model results were like the AdaBoost in estimating the CS of GPCs. The deviation among the experimental and predicted results was in the range of 0.06 to 23.45 MPa, with an average of 5.33 MPa. For 43 mixes, the deviation of the results was less than 3 MPa; deviation from 3 to 10 MPa was observed in 31 mixes, and deviation higher than 10 MPa was observed in only 17 mixes (Figure 16). This analysis further validated the comparable accuracy of the AdaBoost and RF model with higher accuracy than the DT model. Additionally, the higher accuracies of the AdaBoost and RF models was confirmed since, for around 81.3% of mixes, the deviation of the predicted results from the experimental was less than 10 MPa. However, the DT model performed less accurately in estimating the CS of GPCs than the AdaBoost and RF models, as more deviation of results was noted among the experimental and predicted results. Hence, this study recommends the application of AdaBoost and RF models for the prediction of the CS of GPCs.

## 7. Conclusions

The intention of this research was to employ both individual and ensemble machine learning (ML) algorithms to anticipate the compressive strength (CS) of geopolymer composites (GPCs). To forecast outcomes, one individual technique, decision tree (DT), was used, as well as two ensemble techniques, AdaBoost and random forest (RF). The following conclusions have been drawn as a result of this research:

- Ensemble ML approaches (AdaBoost and RF) performed better than the individual ML technique (DT) at predicting the CS of GPCs, with the AdaBoost and RF models performing with a similar degree of precision. The correlation coefficients (R^2^) for the AdaBoost, RF and DT models were 0.90, 0.90, and 0.83, respectively.
- Statistical checks and k-fold analysis verified the model’s performance. Furthermore, these checks also confirmed the comparable accuracy of the AdaBoost and RF models. The lower deviation (MAE, MAPE, and RMSE) of the predicted results and higher R^2^ values of the ensembled models validated their higher precision.
- The comparison of the experimental and predicted results further validated the higher accuracy of AdaBoost and RF models due to less deviation of the predicted results than the experimental results. On the other hand, the deviation of the DT model’s results was higher than the AdaBoost and RF models and is less recommended for estimating the CS of GPCs.
- Sensitivity analysis revealed that fly ash, ground granulated blast furnace slag, and NaOH molarity have a greater influence on the model’s outcome and account for 26.37%, 14.74%, and 13.12% of the contribution, respectively. However, NaOH, water/solids ratio, fine aggregate, gravel 4/10 mm, gravel 10/20 mm, and Na_2_SiO_3_ contributed 11.60%, 9.52%, 7.53%, 6.48%, 5.84%, and 4.80%, respectively, to the prediction of the outcome.
- This type of research will aid the construction sector by enabling the development of quick and cost-effective methods for predicting material strength. Additionally, by promoting eco-friendly construction using these strategies, the acceptance and use of GPC in construction will be expedited.

This study proposes that in upcoming studies, the number of data points and results should be enhanced by experimental research, field trials, and other numerical evaluation techniques (e.g., Monte Carlo simulation). Additionally, to improve the models’ responsiveness, environmental parameters (e.g., elevated/low temperature and humidity) and a detailed description of the raw materials could be incorporated as input factors. Additionally, data from the literature should be retrieved and arranged in such a manner that the influence of different kinds of activators and precursors on the strength of GPCs can be determined using ML techniques.

## Figures and Tables

**Table 1:** Descriptive measurements of input variables employed.

Parameter	Water/Solids Ratio	NaOH Molarity	Gravel 4/10 mm (kg/m^3^)	Gravel 10/20 mm (kg/m^3^)	NaOH (kg/m^3^)	Na_2_SiO_3_ (kg/m^3^)	Fly Ash (kg/m^3^)	GGBS (kg/m^3^)	Fine Aggregate (kg/m^3^)
Minimum	0	1	0	0	3.5	18	0	0	459
Maximum	0.63	20	1293.4	1298	147	342	523	450	1360
Range	0.63	19	1293.4	1298	143.5	324	523	450	901
Median	0.34	9.2	208	789	56	108	120	300	728
Mode	0.53	10	0	0	64	108	0	0	651
Mean	0.34	8.14	288.39	737.37	53.74	111.66	174.34	225.15	729.88
Standard Error	0.01	0.24	19.54	18.82	1.67	2.53	8.82	8.52	6.87
Standard Deviation	0.11	4.56	372.31	358.55	31.91	48.16	167.95	162.27	130.97
Sum	124.8	2955.1	104,684.3	267,664.9	19,508.8	40,532.7	63,286.0	81,728.1	264,947.8

**Table 2:** Statistical assessments of the models employed in this study.

Model	MAE (MPa)	MAPE (%)	RMSE (MPa)
Decision tree	7.016	16.020	10.432
AdaBoost	5.199	12.302	7.467
Random forest	5.325	12.420	7.602

**Table 3:** Outcomes of k-fold analysis.

K-Fold	Decision Tree				AdaBoost				Random Forest			
	MAE	MAPE	RMSE	R^2^	MAE	MAPE	RMSE	R^2^	MAE	MAPE	RMSE	R^2^
1	16.03	18.70	21.92	0.60	8.70	13.25	13.04	0.43	10.70	12.97	13.43	0.54
2	7.02	16.93	11.57	0.76	5.65	12.30	8.01	0.49	5.33	13.76	8.09	0.72
3	9.15	16.03	10.94	0.20	6.56	14.03	8.16	0.79	5.54	14.88	8.37	0.64
4	11.76	17.21	10.43	0.70	8.18	12.55	8.43	0.67	8.06	13.66	11.40	0.52
5	7.31	16.02	12.41	0.59	6.11	12.98	7.47	0.90	5.34	12.90	7.85	0.77
6	12.96	16.55	17.07	0.37	12.94	14.45	14.34	0.57	9.85	13.77	13.82	0.53
7	7.72	18.67	19.58	0.72	9.50	13.66	12.06	0.60	9.43	12.42	15.56	0.74
8	10.92	16.03	15.26	0.41	9.33	13.08	14.33	0.86	11.12	14.02	13.93	0.34
9	8.15	17.22	16.50	0.72	5.20	12.95	7.68	0.74	5.80	13.79	7.60	0.79
10	19.78	17.02	23.23	0.83	14.68	12.35	18.28	0.61	18.47	12.50	19.31	0.90

## Data Availability

Not applicable.

## Supplementary Materials

The following supporting information can be downloaded at: https://www.mdpi.com/article/10.3390/polym14061074/s1, Table S1: Data used for modeling.
